# Manganese exposure and its U-shaped relationship with diabetic retinopathy: analysis of NHANES 2011–2020

**DOI:** 10.3389/fnut.2025.1619751

**Published:** 2025-06-19

**Authors:** Xi Chen, Zhenzhen Gu, Yixin Qi, Xiaofeng Hao, Like Xie

**Affiliations:** Eye Hospital, China Academy of Chinese Medical Sciences, Beijing, China

**Keywords:** manganese, heavy metal, diabetic retinopathy, NHANES, epidemiology

## Abstract

**Background:**

Although many studies have pointed to the role of manganese in various diseases. However, there are surprisingly few studies on the potential relationship between manganese and diabetic retinopathy (DR). The available literature fails to provide definitive conclusions regarding the directionality and strength of this particular association.

**Methods:**

The analytical cohort comprised 2,558 adults from NHANES 2011–2020 cycles. We employed binary logistic regression to evaluate manganese-DR associations, supplemented by subgroup analyses, nonparametric smoothing techniques, and propensity score weighting to address potential confounding.

**Results:**

Our multivariate model failed to find a significant linear relationship between manganese concentration and the likelihood of DR (*p* > 0.05). However, we found that manganese levels above 7.66 μg/L DR were less severe (adjusted OR = 0.84, *p* = 0.0007). This suggests a nonlinear dose–response relationship.

**Conclusion:**

The manganese and DR relationship followed a U-shaped dose–response pattern. The least severe condition was observed at 7.66 μg/L, while the disease was aggravated at both insufficient and excessive concentrations.

## 1 Introduction

DR is a major cause of adult vision loss (1). It involves damage to small blood vessels and nerve cells as well as neovascularization due to chronic hyperglycemia in the body (2). Globally, diabetes affects approximately 537 million people (3). About 103 million adults with diabetes have DR (4). The burden of DR continues to grow. And also from high-income countries to low- and middle-income regions (5). Consequently, combating DR is critical. The pathogenesis of DR primarily stems from long-term diabetes, making glycemic control the cornerstone of its management. Yet it remains highly challenging (6). Growing evidence suggests that trace elements, particularly those involved in antioxidant defense, may modulate DR progression (7–9). Exploring the relationship between micronutrients and DR may help control DR.

Manganese is an important trace element that is widely found in plant foods. It is also effective in preventing inflammation, vascular disease and cancer (10). Manganese acts as a key component of Mn-SOD, the main mitochondrial enzyme responsible for neutralizing superoxide radicals. Therefore, manganese helps relieve oxidative stress and chronic inflammation (11).

It is well known that oxidative Stress as a Major Cause of DR Progression. Experimental studies have shown that Mn-SOD activity attenuates oxidative damage in retinal cells (12). Manganese plays an important role in glucose metabolism (13). Manganese significantly inhibits pro-inflammatory signaling such as TNF-α and IL-11 at moderate concentrations (14). However, while manganese is crucial for health, excessive intake can be harmful (15). Accumulation of excess Manganese may exacerbate oxidative stress via Fenton chemical reaction and and destroys the retinal structure (16). Both manganese deficiency and overload may aggravate oxidative stress and inflammation, worsening DR. While mechanistic understanding has advanced, population-based evidence regarding manganese and DR associations remains limited. Our analysis of NHANES 2011–2020 data seeks to clarify this relationship. Our findings clarify manganese’s dual role in DR pathogenesis, and reveal its potential in diabetes prevention and management.

## 2 Materials and methods

### 2.1 Study participants

**Figure 1:**
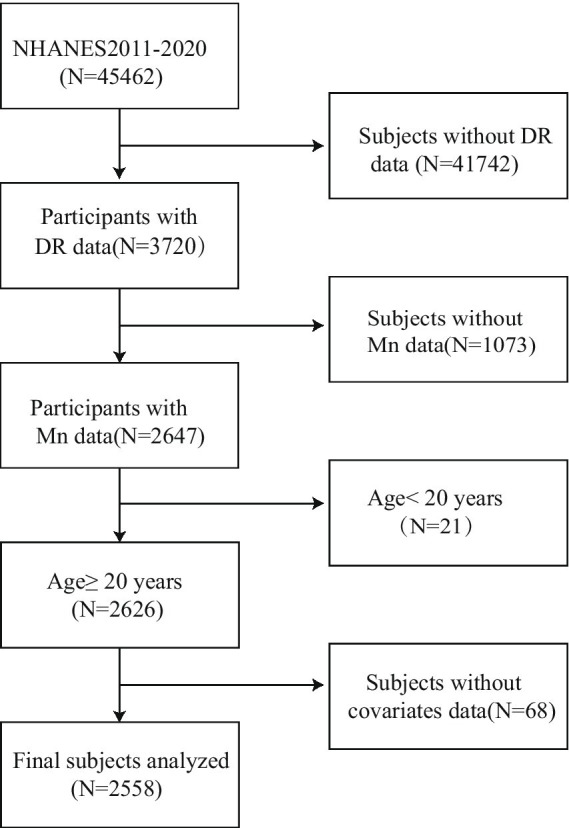
Participant inclusion and exclusion flowchart.

TheNHANES implements a complex, multistage sampling design to generate nationally representative estimates. Our analysis utilized 2011–2020 survey cycles, incorporating both physical examinations and standardized interviews from participating adults. During this period, there were 45,462 participants. We excluded participants with missing DR data (*N* = 41,742) and missing blood manganese data (*N* = 1,073). The final analytical cohort comprised 2,558 eligible adults after excluding minors (*n* = 21) and participants with incomplete covariate data (*n* = 68). The complete participant selection flowchart is presented in Figure 1.

### 2.2 Diabetic retinopathy assessment

Diabetes case identification required meeting at least one of five criteria: (1) HbA1c ≥ 6.5%; (2) Fasting glucose ≥7.0 mmol/L; (3) 2-h OGTT glucose ≥11.1 mmol/L; (4) Self-reported physician-diagnosed diabetes; (5) Current use of glucose-lowering therapy. Among identified diabetics, retinopathy status was determined through standardized self-report (DIQ080), with affirmative responses (“yes”) classifying participants as DR cases.

### 2.3 Determination of blood manganese

Whole blood samples were processed to measure hemorrhagic manganese levels. Measured blood manganese levels are cross-sectional in nature. All blood samples were placed in test tubes containing anticoagulant reagents. They were frozen at −30°C and sent to CDC labs in Atlanta for testing. Before analysis, we mixed samples well to avoid clotting. Whole blood manganese (Mn) concentrations were determined using inductively coupled plasma mass spectrometry (ICP-MS). We diluted them (1:1:48 with water and diluent) before ICP-MS analysis. This method enables high-sensitivity multi-element analysis, ensuring accurate quantification of trace elements. All results met minimum detection limits.

### 2.4 Covariates assessment

Demographic variables comprised sex, age, racial/ethnic background, marital situation, and educational attainment. BMI classifications followed standard cutoffs: under 25 (normal), 25–29.9 (overweight), and 30 + kg/m^2^ (obese). Alcohol consumption was categorized as whether or not they drank more than 4–5 drinks per day based on self-reports. Smoking status was dichotomized into “never smokers” (lifetime smoking <100 cigarettes) and “ever smokers.” Economic status was grouped as “below poverty” (income-to-poverty ratio <1) or “poverty or above” (≥1). Sleep disorders were recorded as present or absent based on self-reports. Participants were considered hypertensive if they met any of these criteria: (1) physician-diagnosed hypertension; (2) current antihypertensive drug use; (3) blood pressure measurements exceeding normal limits.

### 2.5 Statistical analysis

We used weighted statistical methods to improve accuracy. Analyses were performed using weighted multivariate logistic regression models. Independent samples *t*-test was used for continuous variables. Categorical variables were analyzed using chi-square tests. Logistic regression was examined for manganese and DR relationship. Subgroup analyses were conducted using covariates and further stratified multiple regression analyses were conducted to investigate the differences and potential interaction effects between the different subgroups. All analyses were performed using R (version 4.1.1) and EmpowerStats (version 4.2). A *p*-value ≤ 0.05 was considered statistically significant.

## 3 Results

### 3.1 Baseline population profiles

Table 1 presents the study population stratified by DR status. The analysis of DR-related factors revealed that participants with DR had significantly lower educational attainment (*p* = 0.0433). Heavy alcohol consumption (≥4–5 drinks/day) was more prevalent in the DR group (*p* = 0.0208). The prevalence of low educational level and alcohol abuse was higher in the DR group. Highlights the potential role of socioeconomic differences in DR risk. Suggests possible confounders of lifestyle factors. No intergroup variations were detected in lead, cadmium, or mercury levels (*p* > 0.05). However, a significant difference in selenium levels (*p* = 0.0037) was noted, potentially reflecting its antioxidant role in mitigating retinal oxidative damage. No other measured variables demonstrated statistically significant intergroup variations (all *p* > 0.05).

**Table 1:** Baseline characteristics of participants.

Characteristic	No DR	DR	*P*-value
*N*	2,038	520	
Age (years)	61.4 ± 12.8	63.3 ± 12.1	0.8299
Gender (*N*, %)			0.2824
Male	1,082 (52.4)	284 (56.3)	
Female	956 (47.6)	236 (43.7)	
Race (*N*, %)			0.1302
Mexican American	299 (9.4)	68 (8.9)	
Other Hispanic	195 (5.4)	74 (8.4)	
Non-Hispanic White	661 (61.3)	155 (56.2)	
Non-Hispanic Black	577 (13.7)	143 (15.6)	
Other race	306 (10.1)	80 (11.0)	
Marital status (*N*, %)			0.3583
Unmarried or other	834 (37.2)	235 (40.1)	
Married or living with a partner	1,204 (62.8)	285 (59.9)	
Education (*N*, %)			0.0433
Less than high school	584 (20.5)	168 (26.5)	
High school	492 (26.4)	125 (28.9)	
More than high school	962 (53.2)	227 (44.5)	
Smoked at least 100 cigarettes in life (*N*, %)			0.6205
Yes	1,012 (52.2)	245 (50.2)	
No	1,026 (47.8)	275 (49.8)	
4/5 or more drinks per day (*N*, %)			0.0208
Yes	326 (16.3)	91 (19.4)	
No	1,315 (66.6)	314 (56.7)	
Unclear	397 (17.1)	115 (23.9)	
BMI (*N*, %)			0.0699
Normal or low-weight	275 (11.5)	77 (13.8)	
Overweight	584 (26.6)	161 (32.5)	
Obese	1,179 (62.0)	282 (53.7)	
Economic situation (*N*, %)			0.0969
Below poverty	440 (16.1)	124 (21.3)	
Poverty or above	1,368 (75.5)	348 (71.7)	
Unclear	230 (8.4)	48 (6.9)	
Sleep disorder (*N*, %)			0.0941
Yes	757 (38.8)	221 (45.5)	
No	1,281 (61.2)	299 (54.5)	
Hypertension (*N*, %)			0.6353
Yes	1,539 (25.0)	413 (23.6)	
No	499 (75.0)	107 (76.4)	
Lead (ug/dL)	1.4 ± 1.3	1.4 ± 1.3	0.3829
Cadmium (ug/dL)	0.5 ± 0.5	0.5 ± 0.5	0.7814
Mercury (ug/dL)	1.4 ± 2.8	1.4 ± 2.4	0.5513
Selenium (ug/dL)	192.4 ± 30.8	188.3 ± 26.6	0.0037

### 3.2 Manganese and DR association analysis

The nonlinear relationship between Manganese and DR was shown by regression analysis (Table 2). Although the linear model showed no association (*p* > 0.05). However, the tertile analysis revealed a U-shaped dose–response. The risk of DR was significantly lower in T3 participants compared to T1 participants (OR = 0.62, *p* = 0.022). In contrast, the middle tertile (T2) had no significant effect (OR = 1.18, *p* > 0.05). Significant trend tests (p-trend = 0.013–0.015) for all models further confirmed this nonlinear relationship. This protective effect remained stable after multivariable adjustment, suggesting a potential U-shaped relationship between manganese and DR. This suggests that manganese may have a threshold effect and that only optimal levels can be protective against DR.

**Table 2:** The relationship between blood manganese and DR.

Variables	Model 1^a^ β (95% CI) *P*-value	Model 2^b^ β (95% CI) *P*-value	Model 3^c^ β (95% CI) *P*-value
Manganese	0.98 (0.95, 1.01) 0.1868	0.99 (0.98, 1.00) 0.1765	0.99 (0.98, 1.00) 0.2076
Manganese tertile			
T1	Ref.	Ref.	Ref.
T2	1.18 (0.85, 1.65) 0.3240	1.18 (0.84, 1.67) 0.3469	1.20 (0.85, 1.68) 0.3040
T3	0.62 (0.42, 0.93) 0.0225	0.63 (0.42, 0.93) 0.0222	0.62 (0.41, 0.93) 0.0241
P for trend	0.0138	0.0131	0.0154

^a^Non-adjusted model: no adjustment for covariates.

^b^Adjusted model: adjusted for gender age and race.

^c^Adjusted model: adjusted for all covariates.

### 3.3 Identifcation of nonlinear relationship

**Figure 2:**
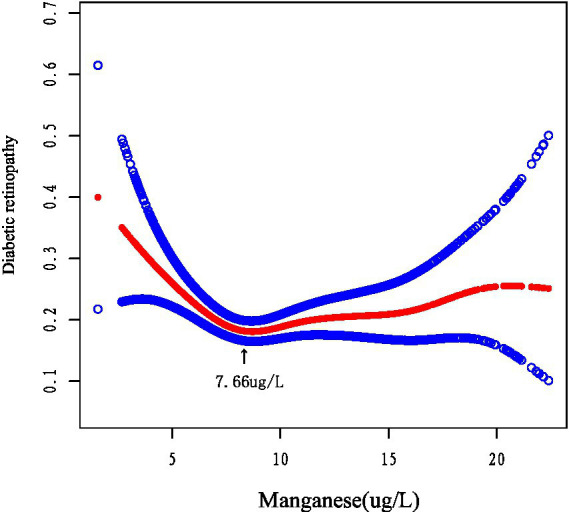
Smoothing curve fitting between manganese and DR.

We using smoothed curve fitting (Figure 2) and segmented regression analysis (Table 3). And nonlinear modeling revealed a U-shaped manganese and DR association. Below 7.66 μg/L, each 1-unit manganese increase conferred 16% lower DR risk (OR = 0.84, *p* < 0.001), while higher concentrations showed null effects (OR = 1.01, *p* = 0.608). The piecewise model significantly outperformed linear regression (*p* = 0.002), indicating concentration-dependent protection.

**Table 3:** Analysis of threshold effects of blood manganese and DR.

Models	Per-unit increase		Per-SD increase	
	OR (95%CI)	*P* value	OR (95%CI)	*P* value
Model 1				
One line effect	0.98 (0.95, 1.01)	0.1513	0.92 (0.83, 1.03)	0.1513
Model 2				
Turning point (K)	7.66		−0.47	
<K	0.84 (0.75, 0.93)	0.0007	0.51 (0.34, 0.75)	0.0007
>K	1.01 (0.98, 1.04)	0.6084	1.03 (0.92, 1.16)	0.6038
95% CI for turning point	−1.64, −1.35		−1.64, −1.35	
P value for LRT test		0.002		0.002

### 3.4 Subgroup analyses

As shown in Table 4, subgroup analyses confirmed the main null association, with no significant Mn and DR relationship observed in any population. This consistency was maintained regardless of age, biological sex, racial/ethnic background, education, marital status, or hypertension (all *p* values > 0.05). Notably, none of the interaction terms reached statistical significance (all *p*-values >0.05), indicating that the manganese and DR relationship was not modified by these demographic or clinical characteristics.

**Table 4:** Subgroup analysis of blood manganese and DR.

Variables	OR(95%CI)	*P* value	P-interaction
Age			0.8704
20–62	0.98 (0.95, 1.02)	0.3386	
63–80	0.98 (0.92, 1.03)	0.4015	
Gender			0.6882
Male	0.99 (0.94, 1.04)	0.6426	
Female	0.98 (0.94, 1.01)	0.2269	
Race			0.9894
Mexican American	1.00 (0.92, 1.08)	0.9653	
Other Hispanic	0.97 (0.88, 1.07)	0.5898	
Non-Hispanic White	0.98 (0.93, 1.03)	0.3954	
Non-Hispanic Black	0.97 (0.90, 1.06)	0.5358	
Other race	0.98 (0.91, 1.05)	0.5268	
Education			0.2430
Less than high school	0.98 (0.93, 1.05)	0.6029	
High school	1.02 (0.96, 1.09)	0.4990	
More than high school	0.96 (0.92, 1.00)	0.0874	
Marital status			0.2115
Unmarried or other	0.95 (0.90, 1.01)	0.0882	
Married or living with partner	1.00 (0.96, 1.04)	0.8146	
Hypertension			0.7903
Yes	0.98 (0.95, 1.02)	0.2930	
No	0.97 (0.91, 1.04)	0.4245	

## 4 Discussion

This study demonstrated for the first time a U-shaped relationship between manganese levels and the risk of DR. The association remained robust after controlling for potential confounders. Our results showed that the likelihood of developing DR was significantly lower when manganese levels reached 7.66 μg/L (OR = 0.84, *p* = 0.0007). These results suggest that maintaining manganese levels around 7.66 μg/L may confer protective benefits. The U-shaped pattern was further confirmed through smoothed curve fitting analysis. There is no globally standardized clinical criteria for a healthy range of blood manganese. The United States Agency for Toxic Substances and Disease Registry (ATSDR) suggests that a blood manganese level of 4–15 μg/L is a common physiologic level. Notably, our data indicate that both excessively high and low manganese levels are associated with increased DR risk. This aligns with existing research demonstrating that elevated manganese concentrations may indeed elevate DR risk (17, 18). Therefore, it may be more appropriate to control blood manganese levels close to 7.66 ug/L. Additionally, the DR group showed significantly lower selenium levels compared to controls (*p* = 0.0037), further supporting the hypothesis that trace nutrient status may influence DR development.

Previous studies have examined associations between serum heavy metals and DR (19). Their findings indicated a significant inverse correlation between manganese and DR, while no significant associations were observed for other heavy metals including selenium. In contrast, our study identified a strong U-shaped relationship between blood manganese levels and DR risk, as well as a significant association between blood selenium levels and DR risk. The reason for this discrepancy may be that they were looking at serum heavy metals from NHANES 2011–2020. However, the NHANES database lacks data on serum selenium and manganese for 2017–2020. By selecting participants with complete blood manganese and selenium data, our results may be more reliable. Although the two studies differ in analytical methods and focus, both highlight the potential role of trace elements in DR pathogenesis. Other research has linked heavy metals to DR risk (20), though manganese was not investigated. Our study specifically addressed this gap by evaluating blood manganese and DR associations. Another study demonstrated that manganese mitigates high-glucose-induced oxidative stress in the retina (21), underscoring its therapeutic relevance. However, the protective effects at specific manganese concentrations remain unclear. Our findings reveal a U-shaped manganese and DR relationship, with levels below the threshold associated with significantly lower DR risk. Additionally, selenium deficiency emerged as a potential risk factor for DR, consistent with existing evidence (22, 23). However, the precise mechanisms by which selenium and manganese influences DR remain unknown. Future studies should further elucidate the roles of these elements in DR prevention and treatment.

Manganese may influence DR through multiple protective mechanisms. First, as an essential cofactor of manganese superoxide dismutase (Mn-SOD). Low concentration of manganese converts superoxide anions (O₂^−^) into H₂O₂ in mitochondria, thereby alleviating hyperglycemia-induced oxidative stress (24, 25). Second, Low concentration of manganese suppresses chronic low-grade inflammation in diabetic retina by reducing NF-κB nuclear translocation and IL-8 release (26–28). Finally, it stabilizes HIF-1α degradation, indirectly inhibiting pathological VEGF overexpression and reducing vascular leakage, thus protecting retinal vasculature (29). However, excessively high blood manganese levels may exacerbate DR. First, High concentration of manganese overload intensifies oxidative stress by accumulating in mitochondria and generating hydroxyl radicals through Fenton reaction, directly damaging retinal pigment epithelial cells (30). Second, High concentration of manganese inhibits tyrosine hydroxylase (TH) activity, reducing retinal dopamine synthesis and impairing microvascular autoregulation (31, 32). Lastly, High concentration of manganese competitively antagonizes selenium/zinc, suppressing selenium-dependent GPx and zinc metalloenzyme activities, thereby weakening coordinated antioxidant defenses (33, 34). In summary, manganese exhibits multifaceted mechanisms affecting DR pathogenesis.

This study has several limitations. First, the cross-sectional design does not provide causal inference of the relationship between Manganese and DR. The directionality of the relationship between Mn exposure and DR is unclear. We can only conclude that there is an association between the two. Thus longitudinal validation is needed. Secondly, determining DR by self-report may introduce bias. Third, because NHANES performs only a single test for diabetes-related indicators. There may be false positives in the diagnosis of diabetes. And it also fails to differentiate the typology of diabetic patients. Finally, although we have tried to be as thorough as possible in analyzing confounding factors that may affect the results of the study. However, due to the presence of unmeasured confounding variables in the database, such as diabetes duration. There are still unmeasured variables that may have influenced. Future studies should incorporate prospective designs, clinical DR assessment, and more extensive covariate collection.

## 5 Conclusion

In this study, the U-shaped relationship between manganese and DR was determined. It was indicating that manganese levels were associated with milder degrees of DR. In particular, moderate concentrations (7.66–12.5 μg/L) of manganese were associated with the mildest degree of DR. In addition, selenium may also reduce DR. It is hoped that future mechanistic studies will elucidate the temporal dynamics of the effects of these micronutrients in order to realize a precision nutritional approach to diabetes prevention.

## Data Availability

The datasets presented in this study can be found in online repositories. The names of the repository/repositories and accession number(s) can be found at: https://wwwn.cdc.gov/nchs/nhanes/default.aspx.
